# Case Report: A Lung Adenocarcinoma With Brain Metastasis Harbored Novel MET 14 Skipping Alteration Sensitive to Savolitinib

**DOI:** 10.3389/fonc.2022.863560

**Published:** 2022-04-04

**Authors:** Jian Li, Yun Feng, Yuan Tan, Qianqian Duan, Qin Zhang

**Affiliations:** ^1^Department of Thoracic Surgery, Shanxi Provincial People’s Hospital, Shanxi, China; ^2^The Medical Department, Jiangsu Simcere Diagnostics Co., Ltd., Nanjing, China; ^3^The Medical Department, Nanjing Simcere Medical Laboratory Science Co., Ltd., Nanjing, China; ^4^The State Key Lab of Translational Medicine and Innovative Drug Development, Jiangsu Simcere Diagnostics Co., Ltd., Nanjing, China

**Keywords:** lung adenocarcinoma, NGS, METex14, Savolitinib, brain metastasis

## Abstract

A splice-site mutation that results in a loss of transcription of exon 14 in the oncogenic driver MET occurs in 3 to 4% of patients with non-small-cell lung cancer (NSCLC). Several MET exon 14 skipping alterations have been identified, but different MET exon splice variants tend to have different clinical outcomes which deserve concern. Herein, based on NGS panel analysis, we firstly described a 61-year-old woman with lung adenocarcinoma who harbored a novel MET exon 14 skipping (c.3004_3028+3del) concurrent MET amplification (copy number: 3.91) and benefited from Savolitinib treatment. Moreover, CytoTest MET/CCP7 FISH Probe (c-MET/CCP7 Ratio:1.41 and mean gene copy number:6) and qPCR which based on ABI 7500 also were performed to confirm these two MET alterations. After 2 months of Savolitinib treatment, the clinical evaluation was a partial response (PR). In summary, our finding not only expanded the spectrum of the MET exon14 variant (METex14). Targeted NGS analysis could improve detection of MET alterations in routine practice.

## Introduction

The mesenchymal–epithelial transition (MET) receptor tyrosine kinase binds the hepatocyte growth factor to activate downstream cell signaling pathways involved in cell proliferation, survival, and migration. Several genetic mechanisms can result in aberrant activation of this receptor in cancer cells. One such activating mechanism involves the acquisition of gene mutations that cause MET exon 14 skipping variants (METex14) during mRNA splicing. Mutations leading to METex14 are found in approximately 3–4% NSCLC patients. Accumulating evidence suggests that METex14 is a true, independent oncogenic driver in NSCLC, and also being an independent prognostic factor for poorer survival in patients with NSCLC (1, 2). Savolitinib is a potent and highly selective oral MET tyrosine-kinase inhibitor and has yielded promising activity and had an acceptable safety profile in patients with NSCLC subtypes positive for METex14 alterations (3). Herein, we report a lung adenocarcinoma with brain metastasis that harbors novel METex14 c.3004_3028+3del alteration concurrent MET amplification and benefits from Savolitinib.

## Case Presentation

**Figure 1:**
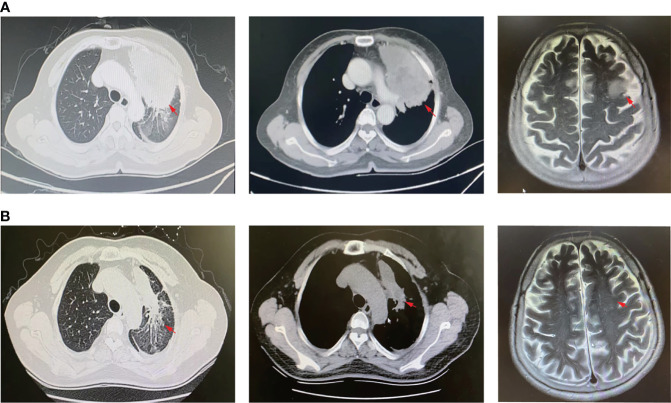
CT scan and MRI images. **(A)** CT scan of lung lesions (left and middle panel) and MRI profile (right panel) before Savolitinib treatment. **(B)** CT scan of lung lesions (left and middle panel) and MRI profile (right panel) 2-months post Savolitinib treatment. The red arrow indicates the location of the lesion.

**Figure 2:**
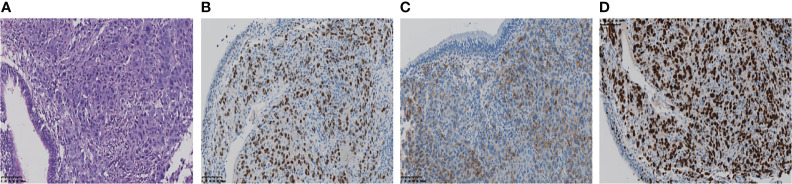
**(A)** The pathological diagnosis of the biopsy was lung adenocarcinoma. **(B, C)** Immunohistochemistry staining: TTF-1, Napsin A, was positive respectively. **(D)** Ki-67 score was 90%.

A 61-year-old woman was admitted to our hospital because of respiratory irritation, dry cough, and breathing difficulty. The patient has no history of smoking, but had occasional alcohol. A computed tomography (CT) scan found a space-occupying lesion in the upper lobe of the left lung. Brain magnetic resonance imaging (MRI) showed left frontal lobe occupation (**Figure 1A**), and she has no history of infectious diseases such as hepatitis, tuberculosis, malaria, and no hypertension, but had a four-year of history of diabetes. A physical examination revealed no lymph node enlargement on both sides of the cervical clavicle, and the chest wall was normal without mass and vascular dilation. Left lung breath sounds clear without pathological murmurs. A percutaneous lung biopsy was performed for immunohistochemical staining, and the results showed a positive expression in thyroid transcription factor-1 (TTF-1) and Napsin A, but negative expression in cytokeratin 5/6 (CK5/6), and the Ki-67 score was 90% (**Figure 2**). Subsequently, an integrated analysis of clinical and pathological results indicated a stage IV (cT4NxM1) poorly differentiated adenocarcinoma.

**Figure 3:**
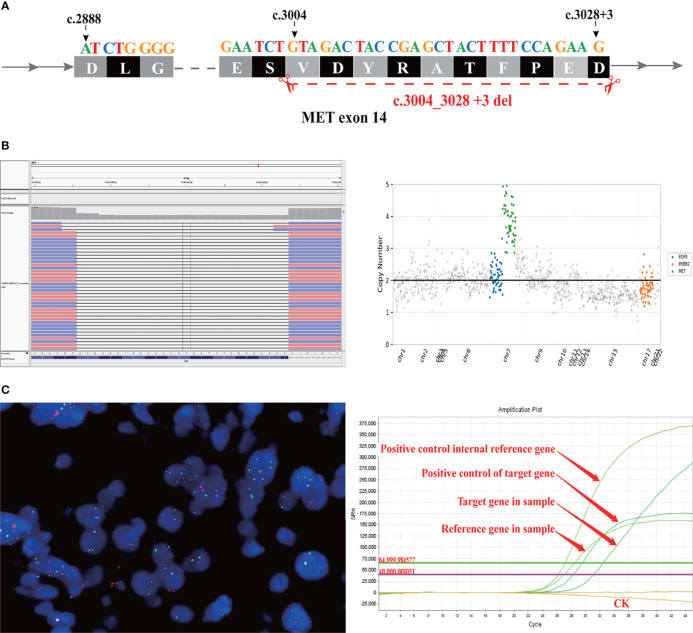
A novel MET exon 14 skipping variant concomitant MET amplification was discovered in a patient with lung adenocarcinoma. **(A)** Illustration of METex14 variant. **(B)** Sequencing reads of MET is shown by the Integrative Genomics Viewer. **(C)** CytoTest MET/CCP7 FISH Probe results showed MET amplification positive (left panel) and qPCR amplification melt curve based ABI7500 (right panel).

As the patient explicitly refused chemotherapy and sought the possibility of targeted therapy, a next-generation sequencing analysis *via* DNA-based hybrid capture based on gene panel of the lung biopsy was conducted in a CAP certificated laboratory. The qualified DNA libraries were sequenced on an Illumina NovaSeq6000 platform (Illumina, San Diego, CA) and generated 150 bp paired-end reads. Then base calls from the Illumina NovaSeq6000 were conducted to FASTQ files. Then a BWA-MEM (v.0.7.17) algorithm was performed to align to the reference genome (UCSC’s hg19 GRCh37). SNVs/InDels were called and annotated *via* VarDict (v.1.5.7) (4) and InterVar (5), then the variants were filtered against the common SNPs in the public database, namely, the 1000 Genome Project and Exome Aggregation Consortium (ExAC) Browser28 (v.0.3), and CNVs were analyzed by the CNVkit (dx1.1) (6). A novel MET14 c.3004_3028+3del variant (mutation abundance: 59.88%) was identified in this patient (**Figure 3A**). Bioinformatic analysis predicted that this mutation might lead to splice of the MET 14 exon as a functionally activated mutation. Interestingly, this patient also harbored MET amplification (copy number: 3.91) (**Figure 3B**). Moreover, we also performed FISH (c-MET/CCP7 Ratio:1.41, mean c-MET copy number per cell:6) and qPCR to validate the MET amplification and METex14 (**Figure 3C**). Based on these robust results, this patient was given Savolitinib 600 mg daily. After 2 months of Savolitinib treatment, shortness of breath and cough of the patient were relieved. There were no other drug-related side effects except mild edema of the lower extremities and the CT showed that the volume of lung and brain lesions was significantly reduced than baseline and the clinical evaluation was a partial response (PR) (**Figure 1B**). The patient was continued to be treated with Savolitinib at the same dose. After 5 months of follow-up, by the time of manuscript submission, the patient was in good condition without progress, and clinical follow-up would continue for this patient.

## Discussion

A splice-site mutation that results in a loss of transcription of exon 14 in the oncogenic driver MET occurs in patients with NSCLC. MET exon 14 alteration has been implicated as an oncogenic driver in NSCLC and has been proposed as a potential therapeutic target (7). Although various METex14 had been identified in several studies (2, 8), the MET c.3004_3028 +3del have not been reported. In this case, to our knowledge, it is firstly describing a novel MET c.3004_3028+3del alteration in a lung adenocarcinoma with brain metastasis patient who showed dramatic response to Savolitinib.

Interestingly, we found that this patient also harbored MET amplification. Since the definition of MET amplification and high copy number amplification based on NGS is still controversial, we performed the MET/CCP7 FISH probe to validate the amplification which showed that the c-MET/CCP7 ratio = 1.41, mean c-MET gene copy number/cell = 6. Previous retrospective studies demonstrated that patients with METex14 both with and without MET amplification have responded to MET TKIs (9, 10). Mark et al. (8) described a case about a lung adenocarcinoma patient harboring c.3028G>A mutation concurrent high-level amplification who showed dramatic response to Crizotinib. Equally, a phase II study demonstrated that approximately 10% of METex14 positive NSCLC patients concomitant MET amplification and that such patients tend to have a better median progression-free survival (mPFS) and objective response rate (ORR) than patients without MET amplification, although not statistically significant differences. In that study, only 5 METex14 NSCLC patients concomitant MET amplification, and no specific description of the molecular information of these patients (3). Moreover, in previous molecular alterations landscape research, there are other concurrent somatic mutations in METex14 positive patient, for instance, the common co-mutated genes are TP53, NF1 and so on (2, 11). Whether overall there is a differential response to MET TKIs according to MET amplification status or concurrent other somatic mutations in the context of METex14 alterations need to be investigated in a larger series of patients or clinical trials to find out the underlying mechanism.

Brain metastases (BrMs) are associated with significant morbidity and are found in up to 50% of patients with advanced NSCLC. It has been recognized that patients with brain metastases comprise a heterogeneous population. With regard to brain metastases from NSCLC, the disease-specific graded prognostic assessment (GPA) has identified patient groups for which median survival ranges from 3 to 14.8 months (12). Clinical trials of systemic treatments largely exclude patients with BrMs. Chemotherapy is an active treatment for BrM with response rates in the brain similar to other sites of disease. Targeted systemic treatments in patients with driver mutations (EGFR and ALK-MET to date) have impressive central nervous system (CNS) penetrance and response rates (13, 14). Some clinical trials enrolled METex14 positive patients with BrMs as well, for instance, among the 11 patients with brain metastases of the VISION study the objective response rate (ORR) was 55% (95% CI, 23 to 83), the median duration of progression-free survival (mPFS) was 10.9 months (95% CI, 8.0 to could not be estimated) (7). Analogously, in a phase II study of Savolitinib, the 15 patients with brain metastases showed stable or decreased brain lesions after Savolitinib treatment [ORR: 46.7% (95% CI, 21.2 to 73.4); mPFS:6.7 month (95% CI, 8.0 to could not be estimated)] (3). In this case, after two months of Savolitinib treatment, the lesions of the lung and brain were significantly reduced than baseline, the clinical evaluation was a partial response (PR).

However, there are also some limitations in our present study. There is only a one-patient case report. Whether sensitivity to Savolitinib in this patient was conferred more by the METex14 mutation or by MET amplification is unclear. Prospective clinical trials will be necessary to determine if certain METex14 mutations are more responsive to c-Met inhibition than others and whether concurrent MET amplification predicts increased sensitivity to c-Met inhibitors. Secondly, more prospective clinical trials are urgent to analyze the efficiency of MET-TKI in MET14ex alterations positive patients with BrMs. In summary, our case report expands the spectrum of METex14 skipping alterations. A targeted NGS analysis could improve the detection of METex14 mutations and MET amplification in routine practice.

## Conclusion

We firstly described a lung adenocarcinoma patient harboring a novel MET c.3004_3028+3del benefited from Savolitinib treatment. Our case report expanded the spectrum of METex14. A targeted NGS analysis could improve detection of MET alterations (mutation, amplification, fusion, and so on) in routine practice. The opportunity to implement precision medicine in oncology has grown in parallel with the increasing use of NGS. This case is an excellent example.

## Data Availability Statement

The original contributions presented in the study are included in the article/supplementary material. Further inquiries can be directed to the corresponding author.

## Ethics Statement

Written informed consent was obtained from the individual(s) for the publication of any potentially identifiable images or data included in this article.

## Author Contributions

JL, YT, QZ, and QQD prepared the manuscript and the literature search. JL and YF reviewed and edited the manuscript. JL treated and observed the patient. JL performed the histopathological, immunohistochemical examinations. All authors listed have made a substantial, direct, and intellectual contribution to the work and approved it for publication.

## Conflict of Interest

YT, QQD, and QZ are employed by Jiangsu Simcere Diagnostics Co., Ltd. and Nanjing Simcere Medical Laboratory Science Co., Ltd.

The remaining authors declare that the research was conducted in the absence of any commercial or financial relationships that could be construed as a potential conflict of interest.

## Publisher’s Note

All claims expressed in this article are solely those of the authors and do not necessarily represent those of their affiliated organizations, or those of the publisher, the editors and the reviewers. Any product that may be evaluated in this article, or claim that may be made by its manufacturer, is not guaranteed or endorsed by the publisher.

## Abbreviations

METex14, MET exon 14 skipping; NSCLC, non-small cell lung cancer; MET, mesenchymal–epithelial transition; mPFS, median progression free survival; ORR, objective response rate.
